# HCF1 and OCT2 Cooperate with EBNA1 To Enhance OriP-Dependent Transcription and Episome Maintenance of Latent Epstein-Barr Virus

**DOI:** 10.1128/JVI.00239-16

**Published:** 2016-05-12

**Authors:** Jayaraju Dheekollu, Andreas Wiedmer, Daniel Sentana-Lledo, Joel Cassel, Troy Messick, Paul M. Lieberman

**Affiliations:** The Wistar Institute, Philadelphia, Pennsylvania, USA

## Abstract

Epstein-Barr virus (EBV) establishes latent infections as multicopy episomes with complex patterns of viral gene transcription and chromatin structure. The EBV origin of plasmid replication (OriP) has been implicated as a critical control element for viral transcription, as well as viral DNA replication and episome maintenance. Here, we examine cellular factors that bind OriP and regulate histone modification, transcription regulation, and episome maintenance. We found that OriP is enriched for histone H3 lysine 4 (H3K4) methylation in multiple cell types and latency types. Host cell factor 1 (HCF1), a component of the mixed-lineage leukemia (MLL) histone methyltransferase complex, and transcription factor OCT2 (octamer-binding transcription factor 2) bound cooperatively with EBNA1 (Epstein-Barr virus nuclear antigen 1) at OriP. Depletion of OCT2 or HCF1 deregulated latency transcription and histone modifications at OriP, as well as the OriP-regulated latency type-dependent C promoter (Cp) and Q promoter (Qp). HCF1 depletion led to a loss of histone H3K4me3 (trimethylation of histone H3 at lysine 4) and H3 acetylation at Cp in type III latency and Qp in type I latency, as well as an increase in heterochromatic H3K9me3 at these sites. HCF1 depletion resulted in the loss of EBV episomes from Burkitt's lymphoma cells with type I latency and reactivation from lymphoblastoid cells (LCLs) with type III latency. These findings indicate that HCF1 and OCT2 function at OriP to regulate viral transcription, histone modifications, and episome maintenance. As HCF1 is best known for its function in herpes simplex virus 1 (HSV-1) immediate early gene transcription, our findings suggest that EBV latency transcription shares unexpected features with HSV gene regulation.

**IMPORTANCE** EBV latency is associated with several human cancers. Viral latent cycle gene expression is regulated by the epigenetic control of the OriP enhancer region. Here, we show that cellular factors OCT2 and HCF1 bind OriP in association with EBNA1 to maintain elevated histone H3K4me3 and transcriptional enhancer function. HCF1 is known as a transcriptional coactivator of herpes simplex virus (HSV) immediate early (IE) transcription, suggesting that OriP enhancer shares aspects of HSV IE transcription control.

## INTRODUCTION

Epstein-Barr virus (EBV) is a human gammaherpesvirus that has been etiologically linked to diverse malignancies, including Burkitt's lymphoma, nasopharyngeal carcinoma, and AIDS-associated non-Hodgkin's lymphomas (1, 2). Like all herpesviruses, EBV establishes a lifelong latent infection that periodically reactivates. Latent infection is observed in long-lived memory B lymphocytes, as well as in EBV-associated tumors. During latency, EBV expresses a limited number of viral genes to maintain a latent state. However, the pattern of viral gene expression can vary depending on the host cell or tumor type, and these variations are important for viral persistence and pathogenesis (3).

The different patterns of EBV latent cycle gene expression have been referred to as latency types (4, 5). During immortalization of naive resting B lymphocytes and in the absence of immune surveillance, EBV forms what is referred to as a type III latency, where it expresses the full repertoire of latency genes to drive unlimited host cell proliferation. Cells with EBV in type III latency express the Epstein-Barr virus nuclear antigens (EBNAs) EBNA1, EBNA2, EBNA3A, EBNA3B, EBNA3C, EBNA-LP (LP stands for leader protein), the latency membrane proteins LMP1, LMP2A, and LMP2B, the Bcl2 homologue BHRF1, the polymerase III (Pol III)-transcribed EBV-encoded small RNAs EBERs 1 and 2, the long noncoding RNAs initiating from the RPMS1 promoter and numerous microRNAs (miRNAs). In Burkitt's lymphoma (BL) and in proliferating memory B cells, EBV establishes a type I latency where only EBNA1 mRNA and noncoding RNAs are expressed. Epithelial cell-derived nasopharyngeal carcinomas (NPCs) and gastric carcinomas (GCs) establish a type II latency where EBNA1 and LMP1 genes, along with noncoding RNA, are expressed. Variations of these transcription patterns have been observed in different tumor types.

In proliferating tumor cells, EBV latency requires the consistent expression of EBNA1. EBNA1 is a sequence-specific DNA-binding protein that binds to multiple repeat elements in the viral origin of plasmid replication (OriP) (6). EBNA1 binding to OriP is required for initiation of DNA replication, episome maintenance, and transcriptional activation of other viral genes, including EBNA2 and LMP1 (7). EBNA1 binds DNA through its C-terminal domain that shares structural similarity to the Kaposi's sarcoma-associated herpesvirus (KSHV) LANA and the human papillomavirus (HPV) E2 proteins (8). EBNA1 is known to interact with several host cell proteins, including importin α, nucleolin, EBP2, HAUSP7, CKII, PML, BRD2, and GMP synthase (9–12).

EBNA1 is required to activate transcription of EBNA2 during the establishment of latent infection and immortalization of naive B lymphocytes (13). EBNA1 is known to function as a transcriptional enhancer when bound to the family of repeats (FR) within OriP (14). The chromatin structure of OriP is also known to have complex features, including nucleosome-free regions at the EBNA1 binding sites (15), positioned nucleosomes at the dyad symmetry (DS) boundaries (16, 17), and cell cycle changes to histone modifications (18–20). More-recent studies have shown that OriP can physically interact through DNA looping with the C promoter (Cp) or LMP1/2 control region in type III latency (20), or alternatively with the Q promoter (Qp) in type I latency (21). Qp is an alternative promoter for generating the EBNA1 transcript in type I latency when the Cp is epigenetically silenced. How OriP functions as a transcriptional enhancer that selectively engages one or the other viral promoters and whether this is linked to other sequence-specific transcription factors, histone modifications, and modifying enzymes is presently unknown.

Several cellular factors have been implicated in binding with EBNA1 to OriP and contribute to the EBNA1-dependent enhancer function. At the DS element, telomere repeat factors (TRFs) bind to three nonamer elements that flank EBNA1 binding sites (22). These TRF binding sites are important for DS-mediated DNA replication and OriP-dependent episome maintenance but have not been implicated in transcriptional regulation (23). At the FR, octamer-binding transcription factor 2 (OCT2), ARID3A, and E2F-1 have been implicated in the EBNA1-dependent transcriptional regulation of Cp (24, 25). OCT2 has been implicated in binding to FR, but it is not clear if this binding is direct or partially dependent on cofactors (24). OCT proteins are known to interact with several cofactors that can modify DNA binding and transcription function. For example, OCT1 binds to the herpes simplex virus (HSV-1) virion component VP16 to activate viral immediate early gene transcription (26, 27). VP16, in turn, recruits other cellular proteins, including host cell factor 1 (HCF1), which is also involved in the transcriptional activation of host cell cycle genes (27, 28). HCF1 associates with several chromatin-modifying complexes, including the histone H3 lysine 4 (H3K4) methyltransferases (mixed-lineage leukemia [MLL] and SETD1), histone demethylases (KDM1A and PHF8), histone acetyltransferase (HAT) KAT8, histone deacetylase (HDAC) SIN3A, *O*-glycosyltransferase OGT, ubiquitin hydrolase RNF2 (BAP-1), and the phosphatase PPA1 (29). How host cell factors, like OCT2 and HCF1, may function with EBNA1 to modify OriP chromatin and regulate EBV latency gene transcripts and whether this resembles mechanisms used by HSV-1 are not yet known. Here, we present evidence that EBNA1 and OCT2 bind cooperatively at FR and recruit HCF1 to stimulate latency gene transcription and maintain episome stability during latency.

## MATERIALS AND METHODS

### Cells, plasmids, shRNAs, and antibodies.

EBV-positive Burkitt's lymphoma cells (MutuI, Raji, SavI, and KemI) and a MutuI virus-derived lymphoblastoid cell line (LCL) were grown in RPMI 1640 medium (Gibco BRL) containing 15% fetal bovine serum and antibiotics penicillin and streptomycin (50 U/ml). HEK 293T cells were culture in Dulbecco's modified Eagle's medium (DMEM) with 10% fetal bovine serum and antibiotics. All the cells were cultured at 37°C and 5% CO_2_ environment. Bacterial expression plasmids for OCT2 and EBNA amino acids (aa) 428 to 619 were constructed by cloning into the BamHI (5′) and SalI (3′) sites of PET 28(-) His vector. Small hairpin RNAs (shRNAs) for OCT2 (shOCT2), host cell factor 1 (shHCF1) and the control (shControl) were obtained from the Sigma/TRC (The RNAi Consortium) collection of targeted shRNA plasmid library (TRC no. 20819, 16263, and 157211). The shEBNA1 was made by cloning small hairpin RNA into the same lentiviral vector as in the Sigma/TRC shRNA library. Lentivirus particles were generated in 293T-derived packaging cell lines. The plasmids containing FLAG-tagged EBNA1 (f-EBNA1) have been described previously (30). T7 HCF1 expression plasmids were described previously (31) (gift of Angus Wilson). Rabbit polyclonal anti-HCF1 (catalog no. A301-400A; Bethyl), mouse monoclonal anti-OCT2 (catalog no. 395400; Invitrogen), mouse monoclonal antiactin (catalog no. A3854; Sigma), mouse monoclonal anti-T7 (catalog no. 69522; Novagen) mouse monoclonal anti-EBNA1 (catalog no. BM3127; Acris), rat anti-EBNA2 (catalog no. 50175912; Fisher), anti-mouse anti-LMP1 (catalog no. M0897; Dako), and rabbit polyclonal anti-Zta and anti-EBNA1 antibodies (custom prepared at Pocono Rabbit Farm) were used for Western blotting.

### shRNA-mediated knockdown of EBNA1, OCT2, and HCF1.

EBV-positive cells were infected by spin infection with lentivirus expressing shEBNA1, shOCT2, shHCF1, or shControl shRNA. At 48 h postinfection, 1.0 to 2.5 μg/ml puromycin was added to the media, and cell pools were selected for puromycin resistance for an additional 72 h.

### DNA binding assays.

EBNA1 DNA binding domain (DBD) (aa 459 to 607) was expressed and purified from Escherichia coli as a His-tagged protein. OCT2 DBD (aa 175 to 345) was expressed and purified as a His-SUMO (small ubiquitin-like modifier) fusion protein. After cleavage with SUMO protease, untagged protein was further purified by conventional chromatography. Proteins were estimated to be >90% pure after gel filtration chromatography. Electrophoresis mobility shift assay (EMSA) with EBNA1 has been described previously (32). The homogeneous time resolved fluorescence (HTRF) assay was performed essentially as described previously (33). Double-stranded DNA oligonucleotides with 1×FR (30 bp) or 2×FR (60 bp) were chemically synthesized with 5′ biotin and coupled to streptavidin europium (Eu^3+^) cryptate donor. His-tagged protein was labeled with an allophycocyanin acceptor (XL665) according to the manufacturers' instructions (Cisbio Bioassays). Binding conditions were essentially identical for those described for EBNA1 EMSA reactions. After incubation of protein and DNA for 1 h, the reactions were read for fluorescence resonance energy transfer (FRET) using an Envision detector according to the manufacturers' instructions (PerkinElmer). Similar binding conditions were also employed for the ALPHA screen assay using streptavidin donor beads and nickel chelate acceptor beads according to the manufacturers' instructions (PerkinElmer and reference 34).

### ChIP assays.

Chromatin immunoprecipitation (ChIP) assays were performed as described previously (18). Rabbit polyclonal antibodies for ChIP were either custom generated for EBNA1 (Pocono Rabbit Farm) or were purchased for rabbit and mouse anti-IgG (Santa Cruz Biotechnology), anti-HCF1 (catalog no. A301-400A; Bethyl), anti-RbBp5 (catalog no. A300-109A; Bethyl), anti-Ash2L (catalog no. A300-489A; Bethyl), anti-OCT2 (catalog no RB9297; Neo Markers) and pan-H3 (catalog no. 07-690), H3K4me3 (catalog no. 07-473), H3K9acetyl (catalog no. 07-352) were purchased from Millipore. Rabbit serum anti-H3K9me3 (catalog no. 39161), anti-H3K4me1 (catalog no. 61633), anti-H3K27me3 (catalog no. 39155)- and anti-H3K27acetyl (catalog no. 39685), and anti-H3K4me2 (catalog no. 39141) were from Active Motif.

### EBV episome maintenance by pulsed-field electrophoresis.

MutuI, Raji, SavI, and KemI cells and LCLs were infected with lentivirus as described above. After puromycin selection, cells were resuspended in 1× phosphate-buffered saline (PBS) and an equal amount of 2% agarose to form agarose plugs containing 1 × 10^6^ cells that were then incubated for 48 h at 50°C in lysis buffer (0.2 M EDTA [pH 8.0], 1% sodium lauryl sulfate, 1 mg/ml proteinase K). The agarose plugs were washed twice in TE buffer (10 mM Tris [pH 7.5] and 1 mM EDTA). Pulsed-field gel electrophoresis (PFGE) was performed for 23 h at 14°C with an initial switch time of 60 s and a final switch time of 120 s at 6 V/cm and an included angle of 120° as described previously (Bio-Rad CHEF Mapper) (35). DNA was transferred to nylon membranes by established methods for Southern blotting (36). The DNA was then detected by hybridization with α-^32^P-labeled probe specific for the EBV WP region and visualized with a Typhoon 9410 variable-mode imager (GE Healthcare Life Sciences).

### Immunoprecipitation.

Cells were extracted with lysis buffer (20 mM Tris-HCl [pH 7.4], 1 mM EDTA, 0.1 mM EGTA, 2 mM MgCl_2_, 150 mM NaCl, 1 mM Na_3_VO_4_, 1 mM NaF, 20 mM sodium glycerophosphate, 5% glycerol, 1% Triton X-100, 0.5% sodium dodecyl sulfate, 1× protease inhibitors [Sigma], 1× phosphatase inhibitors [Sigma], and 1 mM phenylmethylsulfonyl fluoride [PMSF]). After rotation for 60 min at 4°C, the lysate was centrifuged for 20 min at 16,000 × *g*, and the supernatant was recovered. The cleared extracts were used for immunoprecipitation with antibodies as indicated in the figures.

### Fluorescent *in situ* hybridization.

MutuI cells were harvested, washed in PBS, and mounted onto slides by cytospin (Shandon Cytospin 3; Thermo Fisher) at 1,000 rpm for 5 min. Fluorescent *in situ* hybridization (FISH) detection of viral DNA was carried out as previously described (37). Images were captured with a 63× lens on a Leica SP5 II confocal microscope (Leica Microsystems) using LAS AF software for image processing and quantification.

### Reverse transcription-quantitative PCR.

Reverse transcription-quantitative PCR (RT-qPCR) assay of viral gene expression was performed as previously described (38).

## RESULTS

### Complex histone modification patterns at OriP.

**FIG 1:**
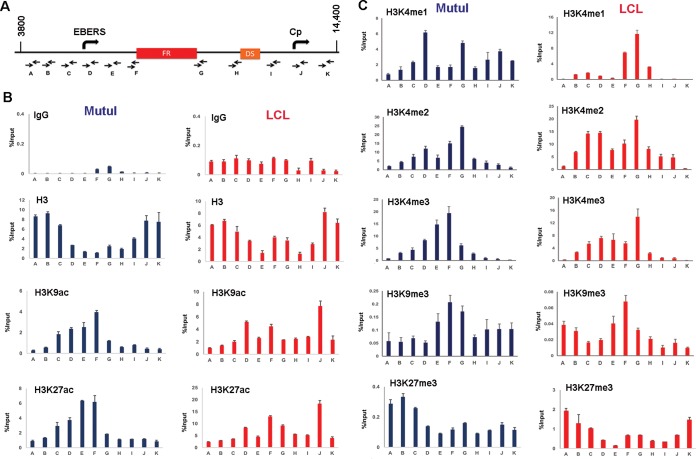
Histone modifications at OriP. (A) Schematic of EBV OriP region and positions of primer sets used for ChIP-qPCR (primers A to K). (B) ChIP-qPCR of histone H3, H3K9ac, H3K27ac, or control IgG in MutuI cells (blue) or LCLs (red). (C) ChIP-qPCR of histones H3K4me1, H3K4me2, H3K4me3, H3K9me3, and H3K27me3 in MutuI cells (blue) or LCLs (red). ChIP DNA is measured as percent input, and error bars represent standard deviations for three technical replicates.

To investigate enhancer-like epigenetic marks of OriP, we analyzed by chromatin immunoprecipitation coupled to detection by quantitative PCR (ChIP-qPCR) the occupancy of several histone modifications in either type I (MutuI) or type III (LCL) latently infected cell lines (Fig. 1). ChIP DNA was assayed using a series of primer pairs spanning an ∼10-kb region encompassing EBV-encoded small RNAs (EBERs), OriP, and the major type III latency promoter Cp (Fig. 1A). We were unable to generate well-behaved PCR primers within the 621-bp repeat region of FR, but used flanking primers (primers F and G) as proxies for the FR region. We first noted that overall histone H3 occupancy was reduced at the regions surrounding FR relative to other regions throughout OriP (Fig. 1B). This is consistent with competitive binding of EBNA1 throughout the FR region (15, 39). Despite the general reduction in total H3 at OriP, we observed a relative enrichment of several histone modifications at the FR boundaries. We found that histone acetylation for H3K9 acetylation (H3K9ac) and H3K27ac was enriched at the EBERs and the 5′ boundary of FR (Fig. 1B). Histone H3K4 methylation was also enriched at regions surrounding FR (Fig. 1C). Enhancer-associated H3K4me1 was strongly enriched at the 3′ end of FR in LCLs (type III), and more noticeably at the EBER promoter region in MutuI (type I) latency. Both di- and trimethylation of H3K4 was enriched through a region spanning EBERs to FR in MutuI cells and LCLs. We observed a low occupancy (0.2% input), but consistent peak of H3K39me3 at FR in both MutuI and LCLs. In contrast, Polycomb-associated H3K27me3 was generally low throughout and mildly suppressed at FR relative to surrounding regions (Fig. 1C). The relative enrichment of euchromatin at OriP is consistent with our previous genome-wide ChIP and sequencing (ChIP-Seq) analyses (20, 40). Taken together, these studies indicate that OriP has complex histone modification patterns, with enhancer-like features, including elevated H3K27ac and H3K4me1, especially in LCLs where OriP is known to enhance Cp transcription.

**FIG 2:**
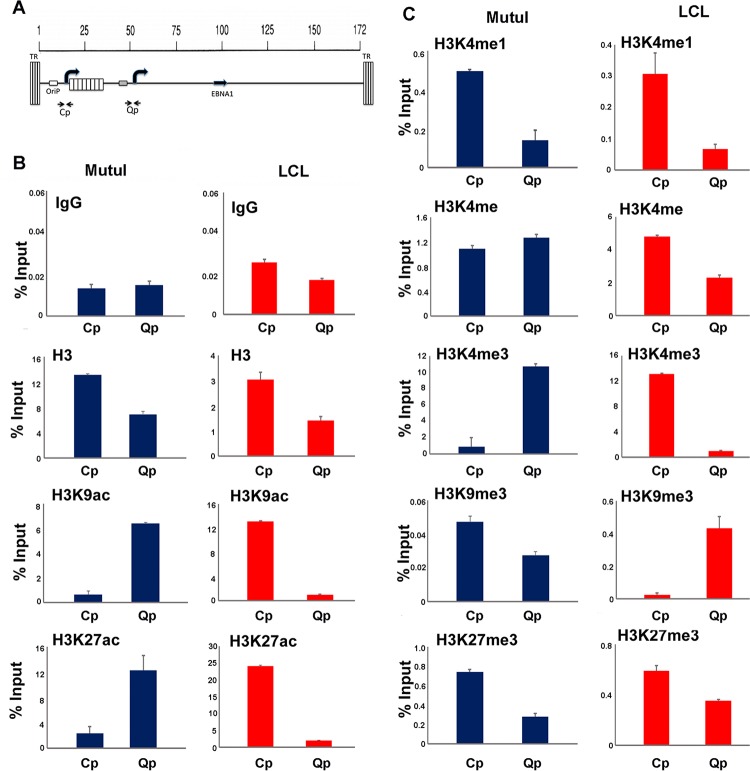
Histone modifications at Cp and Qp. (A) Schematic of Cp and Qp locations on the EBV genome and positions of primer sets used for ChIP-qPCR. TR, terminal repeats. (B) ChIP-qPCR of histone H3, H3K9ac, H3K27ac or control IgG in MutuI cells (blue) or LCLs (red). (C) ChIP-qPCR of histones H3K4me1, H3K4me2, H3K4me3, H3K9me3, H3K27me3 in MutuI cells (blue) or LCLs (red). ChIP DNA is measured as percent input, and error bars represent standard deviations for three technical replicates.

We also examined the histone modification patterns more specifically at the transcription start sites for Cp and Qp, promoters known to be differentially regulated in type III and I latency (Fig. 2A). As expected, histone H3K9ac and H3K27ac were elevated at Qp in type I (MutuI) cells and at Cp in type III (LCLs) (Fig. 2B). A similar pattern of selective enrichment was observed for H3K4me3 (Fig. 2C). Lower levels of enrichment (0.4 to 0.6% input) were observed for histone H3K4me1 at Cp in both MutuI and LCL, while a weak enrichment (0.6% input) of H3K9me3 was observed at Qp in LCLs. These findings indicate that Cp and Qp have different epigenetic marks corresponding to their differential transcription activities in type I and III latencies.

### OCT2 and HCF1 bind throughout the OriP region.

**FIG 3:**
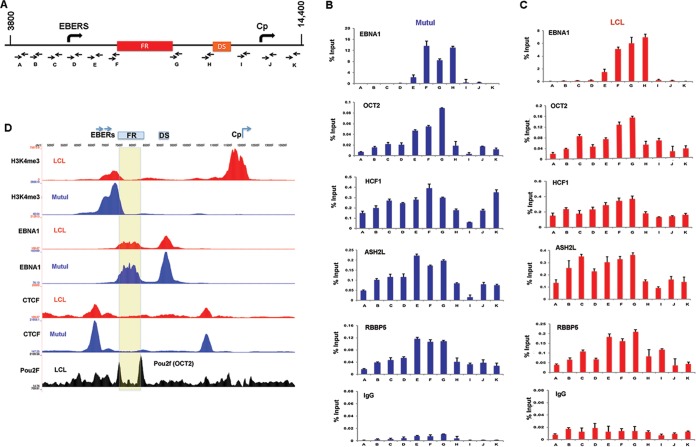
OCT2 and HCF1 are enriched at OriP. (A) Schematic of EBV OriP region and positions of primer sets used for ChIP-qPCR. (B) ChIP-qPCR for EBNA1, OCT2, HCF1, ASH2L, RBBP5, and IgG control in MutuI cells. (C) Same as in panel B, except in LCLs. (D) ChIP-Seq tracks for H3K4me3, EBNA1, or CTCF are shown for LCLs (red) or MutuI cells (blue). OCT2 data set from ENCODE LCLs is shown in black. The FR region is highlighted in yellow.

To identify potential factors that may contribute to OriP enhancer function, we assayed by ChIP-qPCR several candidate cellular factors that have been implicated in FR binding, like sequence-specific DNA-binding proteins EBNA1 and OCT2 or non-DNA-binding chromatin modulators of histone H3K4 methylation, like the MLL complex components HCF1, ASH2L, and RBBP5 (Fig. 3). As expected, EBNA1 bound to FR and DS elements in both MutuI cells and LCLs (Fig. 3B and C). We also found that OCT2 bound weakly (∼0.2% input) to the FR region in both MutuI cells and LCLs, consistent with previous reports that OCT2 functions at the OriP enhancer (24, 25). OCT2 (Pou2f) binding to FR boundaries could also be detected in ChIP-Seq data sets from LCLs studied by the Encyclopedia of DNA Elements (ENCODE) (Fig. 3D). Antibodies to MLL proteins were not efficient at ChIP or Western blotting in our hands. Consequently, we assayed several components of the MLL methyltransferase complex implicated in regulation of H3K4me3 at enhancers. We found that HCF1, ASH2L, and RBBP5 bound as a broad, low occupancy (∼0.2 to 0.5% input) peak across the OriP region, with some variable enrichment at the FR region in both cell types. These findings suggest that MLL or its associated factors may function to regulate histone modifications at OriP.

### OCT2 binds cooperatively with EBNA1 to FR elements in OriP.

**FIG 4:**
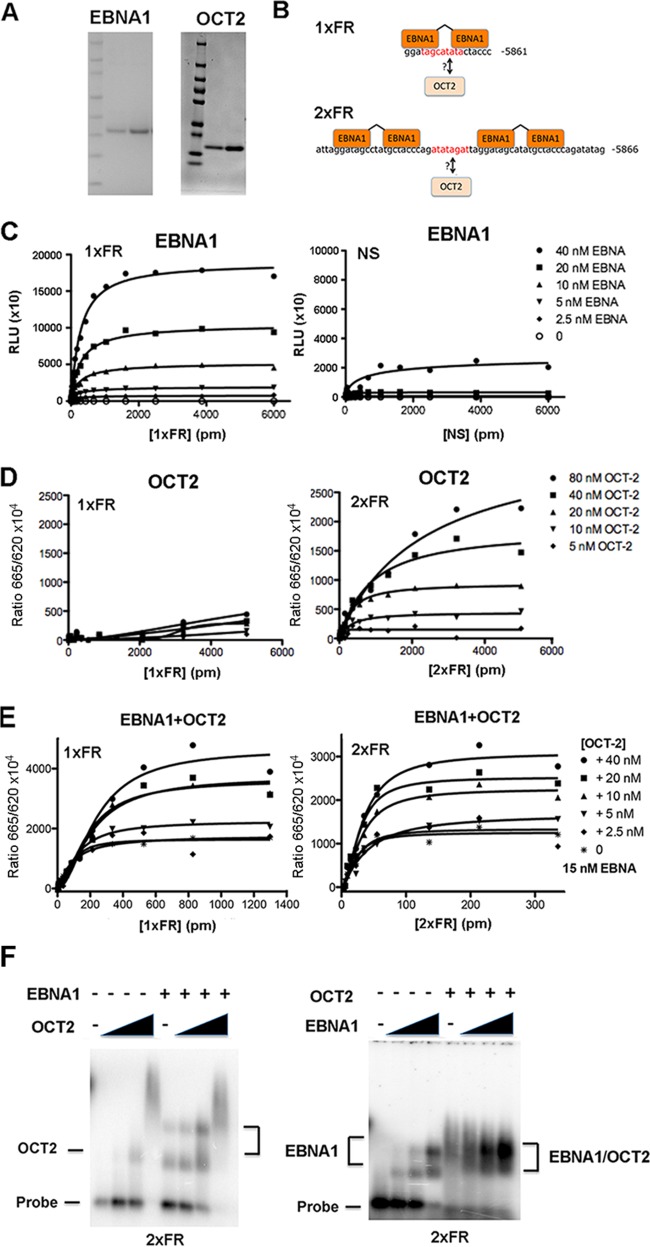
OCT2 binds cooperatively with EBNA1 at FR. (A) Coomassie blue staining and SDS-PAGE of purified EBNA1 DBD and OCT2 DBD. (B) Schematic showing 1×FR or 2×FR probes with EBNA1 monomer recognition sequences and potential OCT2 recognition sequences highlighted in red. (C) ALPHA screen assay with EBNA1 at various concentrations (0 to 40 nM) indicated and probes for either 1×FR or nonspecific DNA (NS) at the concentrations indicated (0 to 6,000 pM). ALPHA signal is indicated in relative light units (RLU). (D) HTRF assay with His-tagged OCT2 (His-OCT2) (0 to 80 nM) with either 1×FR or 2×FR at the concentrations indicated in the figure (0 to 5,000 pM). (E) HTRF assay with 2.5, 5, 10, 20, or 40 nM OCT2 with 15 nM His-EBNA1 using either 1×FR or 2×FR probe at various concentrations as indicated in the figure. The FRET signal is indicated on the *y* axis. (F) EMSA with ^32^P-labeled 2×FR (60-mer) probe with 0, 20, 40, and 80 nM OCT2 DBD without EBNA1 DBD (−) or with 15 nM EBNA1 DBD (+) (left panel) or 0, 5, 10, 2 and 0 nM EBNA1 without OCT2 (−) or with 40 nM OCT2 (+) (right panel). The height of the black triangle indicates the amount of OCT2 DBD (20, 40, and 80 nM).

To investigate the potential binding of OCT2 to FR, we generated highly purified OCT2 and EBNA1 DNA binding domains from E. coli (Fig. 4A) and tested these in various *in vitro* DNA binding assays. We set out to determine whether OCT2 could bind directly to either a single family of repeat (1×FR) or to a tandem pair of repeats (2×FR) and whether this binding was influenced by the presence of EBNA1 (Fig. 4B). We first used an ALPHA screen assay to demonstrate that EBNA1 bound selectively to the 1×FR, but not to a nonspecific control DNA, as expected (Fig. 4C). We then used an HTRF assay to investigate OCT2 DNA binding and found that OCT2 could bind to the 2×FR, but not to the 1×FR (Fig. 4D). We next used the HTRF assay to investigate whether OCT2 and EBNA1 bound cooperatively to either the 1×FR or 2×FR probe (Fig. 4E). We found that the addition of unlabeled OCT2 further stimulated subsaturating amounts of EBNA1 (15 nM) binding to either 1×FR or 2×FR (Fig. 4E), suggesting that OCT2 could bind cooperatively to FR DNA in the presence of EBNA1. We next used agarose gel EMSA to visualize complex formation on the 2×FR probe (Fig. 4F). Addition of increasing concentrations of OCT2 alone formed a single, major species with 2×FR at 40 nM, with some large complex forming at higher concentration (Fig. 4F, left panel). Addition of OCT2 in the presence of 15 nM EBNA1 led to a further stimulation of EBNA1 binding and also formed a large complex at higher concentrations (Fig. 4F, left panel). In the absence of OCT2, EBNA1 bound with low nanomolar affinity to form two major species (Fig. 4F, right panel). In the presence of 40 nM OCT2, EBNA1 bound more FR DNA and produced a saturated complex distinct from EBNA1 alone (Fig. 4F, right panel). Taken together, these *in vitro* DNA binding studies suggest that OCT2 can bind directly to DNA sequence contained between two FR elements (Fig. 4B) and that OCT2 can bind cooperatively with EBNA1 at single FR sites.

### EBNA1 interacts with OCT2 and HCF1.

**FIG 5:**
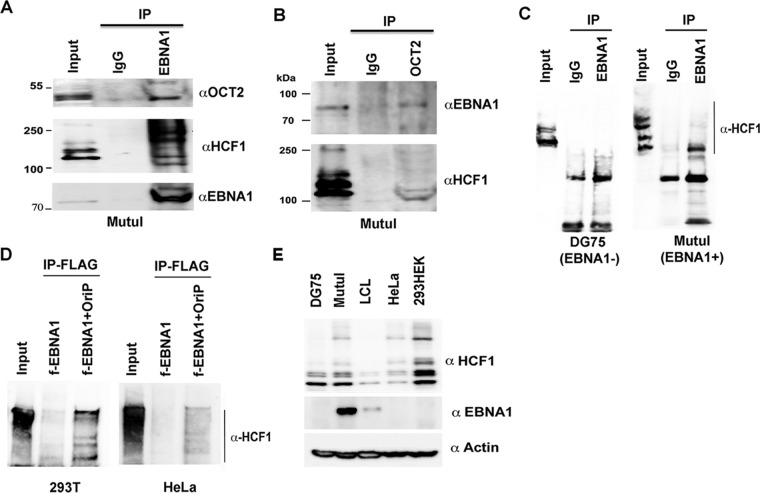
EBNA1 interacts with HCF1 in an OriP-dependent manner. (A) IP with EBNA1 or control IgG from MutuI cells were probed with antibody to OCT2, HCF1, or EBNA1 (αOCT2, αHCF1, or αEBNA1). (B) IP with OCT2 or control IgG from MutuI cells were probed with EBNA1 or HCF1 antibody. (C) EBNA1 or control IgG from DG75 or MutuI cells was assayed with antibody to HCF1. (D) FLAG-tagged EBNA1 (f-EBNA1) was expressed in 293T or HeLa cells with or without OriP plasmid and assayed by FLAG-IP and probed with antibody to HCF1. (E) Western blot of total cell lysates from DG75, MutuI, LCL, HeLa, or HEK 293T cells probed with antibody to HCF1, EBNA1, or actin as indicated in the figure.

To further investigate the potential interactions between EBNA1, OCT2, and HCF1 in living cells, we performed coimmunoprecipitation (coIP) experiments (Fig. 5). We were able to detect OCT2 and multiple mass isoforms of HCF1 in coIPs with EBNA1 antibody using MutuI cell extracts (Fig. 5A). We were also able to detect EBNA1 and isoforms of HCF1 in coIPs with OCT2 antibodies (Fig. 5B). To confirm that the coIP was EBNA1 dependent, we compared EBNA1 IP in EBV-negative DG75 cells to EBV-positive MutuI cells (Fig. 5C). We found that HCF1 coIP could be detected exclusively in EBV-positive MutuI cells, confirming EBNA1 dependence. We next tested the ability of EBNA1 to coIP with HCF1 in EBV-negative 293T cells (Fig. 5D). IP of FLAG-tagged (f-EBNA1) failed to efficiently coIP with HCF1. However, addition of OriP plasmid restored the ability of EBNA1 to coIP with HCF1. This was recapitulated in EBV-negative HeLa cells, suggesting that OriP is required for stable association of EBNA1 with HCF1 (Fig. 5D, right panel). We were unable to detect OCT2 or HCF1 in EBNA1 coIPs from LCL extracts (not shown), but this may be due to the relatively low levels of EBNA1 and HCF1 expressed in these LCLs (Fig. 5E).

### The EBNA1 DNA binding domain is required for interaction with the HCF1 N-terminal domain.

**FIG 6:**
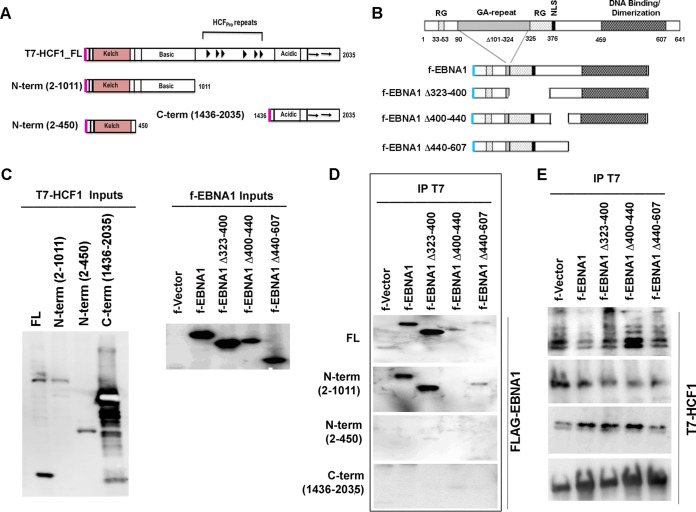
Mapping EBNA1-HCF1 interaction domains. (A) Schematic of HCF1 domains and T7-tagged constructs used for interaction studies. (B) Schematic of EBNA1 domains and FLAG-tagged constructs used for interaction studies. NLS, nuclear localization signal. (C) Input proteins T7-tagged HCF1 (T7-HCF1) and f-EBNA1 were expressed in 293T cells and assayed with either T7 or FLAG antibody. (D) T7-HCF1 proteins were assayed for interaction with f-EBNA1 proteins using T7 IP and assayed with FLAG antibody. All f-EBNA1 plasmid contained OriP DNA. (E) Control for expression of T7-HCF1 proteins used in panel D.

To identify subdomains of HCF1 and EBNA1 important for their interaction, we assayed deletion mutations of each protein in coIP assays (Fig. 6). We assayed T7-tagged full-length HCF1 (FL-HCF1), N-terminal domain Kelch and basic domain (N-terminal domain with amino acids 2 to 1011 [N-term 2-1011]), N-terminal Kelch domain (N-term 2-450), and C-terminal acidic domain (C-term 1436-2035) (Fig. 6A and C). We assayed these for their ability to coIP with FLAG-tagged EBNA1 or deletion mutants of EBNA1 lacking the amino-terminal RG domain (deletion of amino acids 323 to 400 [Δ323-400]), HAUSP7 interaction domain (Δ40-440), or DNA binding and dimerization domain (Δ440-607) (Fig. 6B and C). All EBNA1 expression plasmids also contained OriP, as this was found to be important for HCF1 interactions (Fig. 6D). We detected coIP interactions between FL-HCF1 or N-term (2-1011) with either f-EBNA1 or f-EBNA1 Δ323-400 (Fig. 6D). We detected weak interactions with FL-HCF1 and either f-EBNA1 Δ440-607 or Δ400-440, while no interaction could be observed between HCF1 N-term (2-1011) and f-EBNA1 Δ400-440. No interaction was detectable between HCF1 N-term (2-450) or C-term (1436-2035) with any EBNA1 proteins (Fig. 6D). We were unable to assess any potential interaction of the EBNA1 N-terminal domain (aa 1 to 90), as these mutants were not stably expressed in this plasmid system. All T7-HCF1 proteins were efficiently recovered by T7 IP (Fig. 6E). Taken together, these findings suggest that the HCF1 N-terminal domain containing aa 2 to 1011 is required for interaction with EBNA1 domains that include regions spanning aa 400 to 607, encompassing both the DNA binding domain and HAUSP7 interaction domain.

### OCT2 and HCF1 are required for transcriptional control of EBV latent cycle genes.

**FIG 7:**
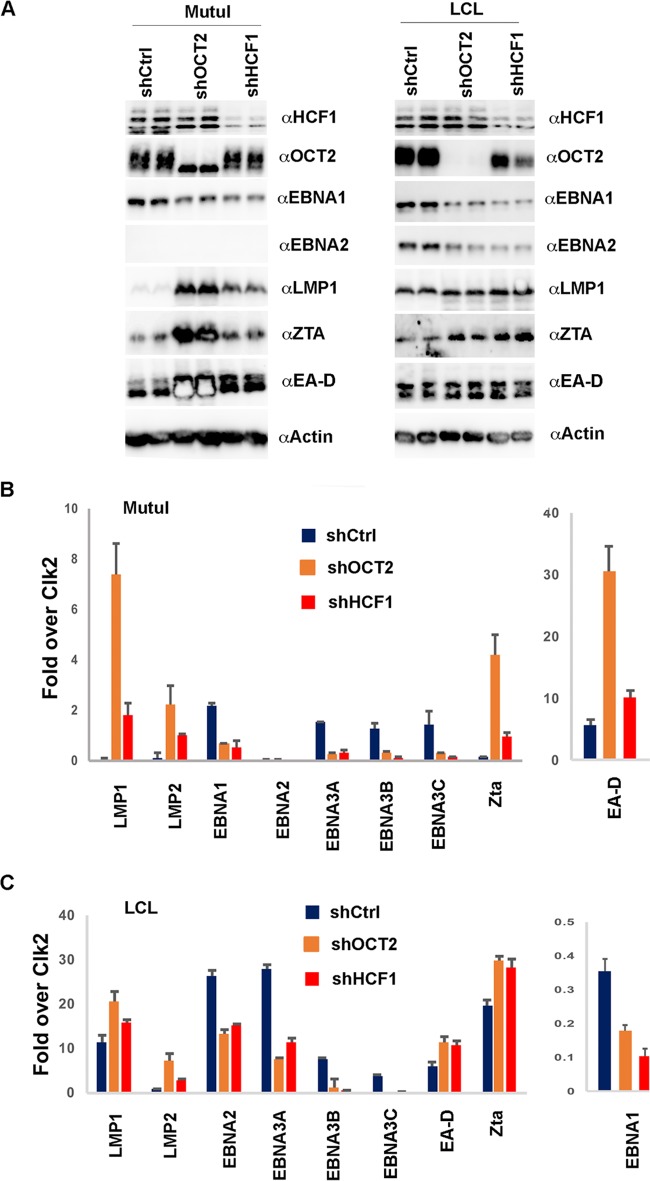
Depletion of OCT2 and HCF1 deregulates EBV latent cycle transcription. (A) Western blots of MutuI cells or LCLs transduced with shCtrl, shOCT2, or shHCF1 and probed with antibody to OCT2, HCF1, EBNA1, EBNA2, LMP1, Zta, or actin as indicated in the figure. (B) RT-qPCR for EBV genes (as indicated) in MutuI cells transduced with shCtrl, shOCT2, or shHCF1. (C) Same as in panel B, except in LCLs.

To assess the functional contribution of OCT2 and HCF1 to EBV latent cycle gene expression, we used lentivirus-mediated shRNA knockdown in LCLs and MutuI cells (Fig. 7). We found that OCT2 and HCF1 depletion was evident in both cell types (Fig. 7A). In MutuI cells, both OCT2 and HCF1 depletion led to a modest decrease in EBNA1 expression and an increase in LMP1. In MutuI cells, OCT2 depletion led to an increase in Zta and EA-D expression (Fig. 7A), consistent with its reported role in repressing lytic cycle reactivation (41). In LCLs, OCT2 and HCF1 depletion resulted in a decrease in EBNA1 and EBNA2 expression and a modest increase in LMP1 and Zta expression. RT-PCR analysis revealed similar trends in viral gene expression (Fig. 7B and C). In MutuI cells, depletion of OCT2 and HCF1 led to a decrease in EBNA1, while depletion of OCT2 led to a more significant increase in LMP1, LMP2, and lytic cycle genes Zta and EA-D (Fig. 7B). In LCLs, depletion of OCT2 and HCF1 led to a reduction in EBNA1, EBNA2, EBNA3A, -3B, and -3C transcripts, with a modest increase in LMP1, LMP2, and Zta (Fig. 7C). These findings indicate that OCT2 and HCF1 have complex functional roles in regulating EBV latency gene expression, including the activation of latency genes and suppression of lytic cycle gene activation.

### HCF1 is required for histone modification patterning at the OriP enhancer and Cp or Qp promoter.

**FIG 8:**
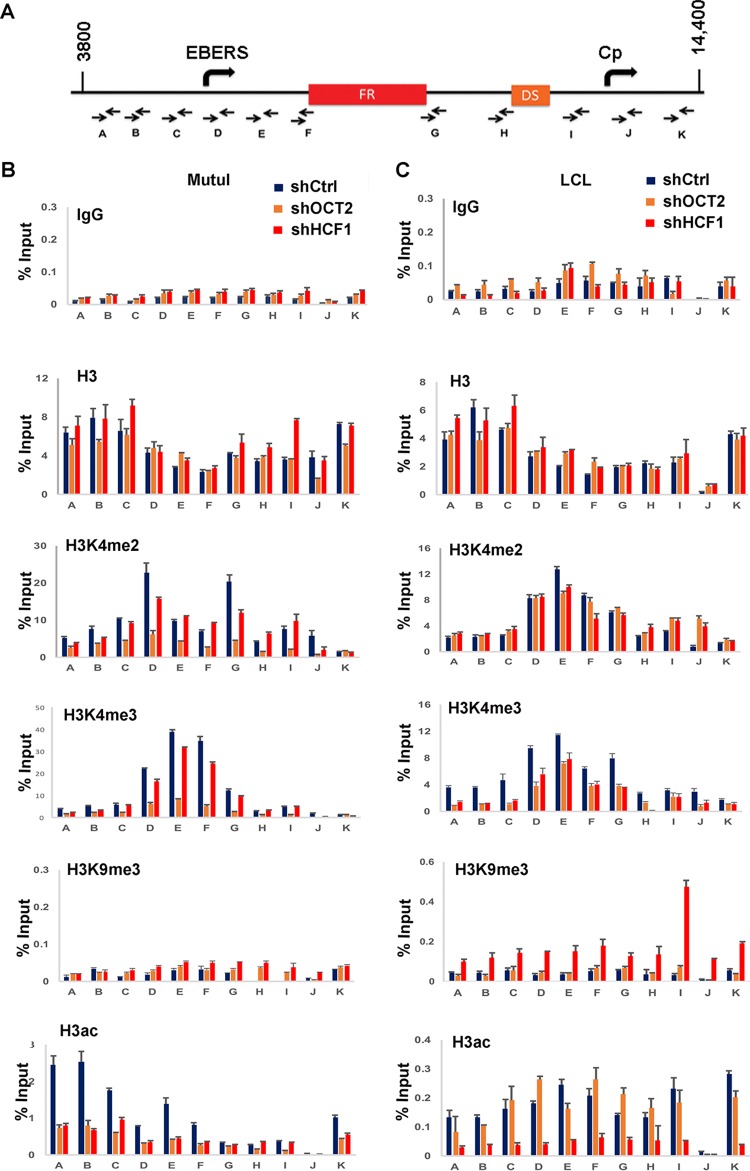
Depletion of OCT2 and HCF1 deregulate histone modifications at the OriP locus. (A) Schematic of primer positions used for qPCR. (B) ChIP-qPCR for IgG, histone H3, H3K4me2, H3K4me3, H3K9me3, or H3ac in MutuI cells transduced with shCtrl, shOCT2, or shHCF1. Primer positions are indicated below each graph. (C) Same as in panel B, except in LCLs.

To investigate the mechanism of HCF1 and OCT2 in regulating the OriP enhancer, we assayed the effects of OCT2 and HCF1 depletion on histone modifications at the OriP locus (Fig. 8). Depletion of OCT2 led to a reduction in H3K4me2 and H3K4me3 at the EBER and FR regions in MutuI cells, and to a lesser extent in LCLs. In LCLs, HCF1 depletion led to an increase in H3K9me3 throughout OriP and the Cp region. OCT2 and HCF1 depletion led to a substantial loss of acetylated H3 (H3ac) in MutuI cells, while only HCF1 depletion led to a loss of H3ac in LCLs. These findings underscore the significant but complex role of OCT2 and HCF1 in regulating histone modifications at the OriP locus.

**FIG 9:**
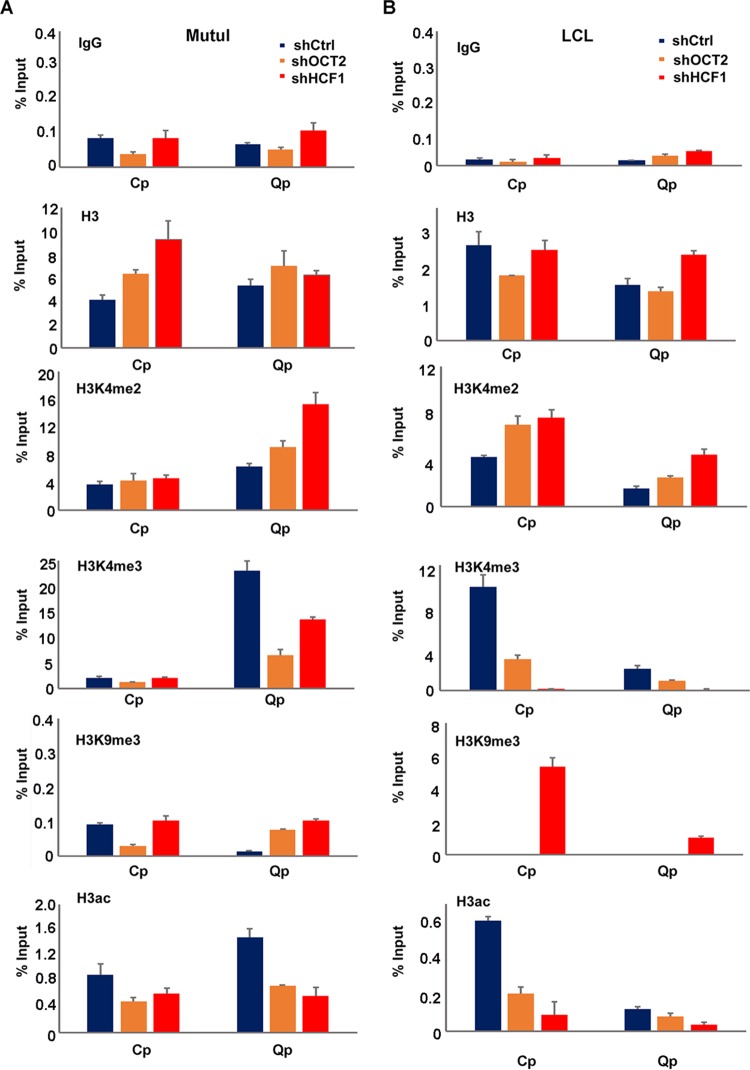
Depletion of OCT2 and HCF1 deregulates histone modifications at Cp and Qp. (A) ChIP-qPCR for IgG, histone H3, H3K4me2, H3K4me3, H3K9me3, or H3ac in MutuI cells transduced with shCtrl, shOCT2, or shHCF1 at Qp and Cp. (B) Same as in panel A, except in LCLs.

We also examined the effects of HCF1 and OCT2 depletion on the Cp and Qp promoter regions more specifically (Fig. 9). In MutuI cells where Qp is more active, we found that depletion of either OCT2 or HCF1 reduced the enrichment of H3K4me3 and H3ac at Qp. In LCL cells where Cp is more active, HCF1 and OCT2 depletion lead to a loss of H3K4me3 and H3ac at Cp. We also observed an increase in H3K9me3 at Cp, as well as at Qp in HCF1-depleted LCLs. These findings indicate that HCF1 and OCT2 function to maintain the epigenetic state of the activated promoter for each latency type.

### HCF1 is required for EBV episome maintenance.

**FIG 10:**
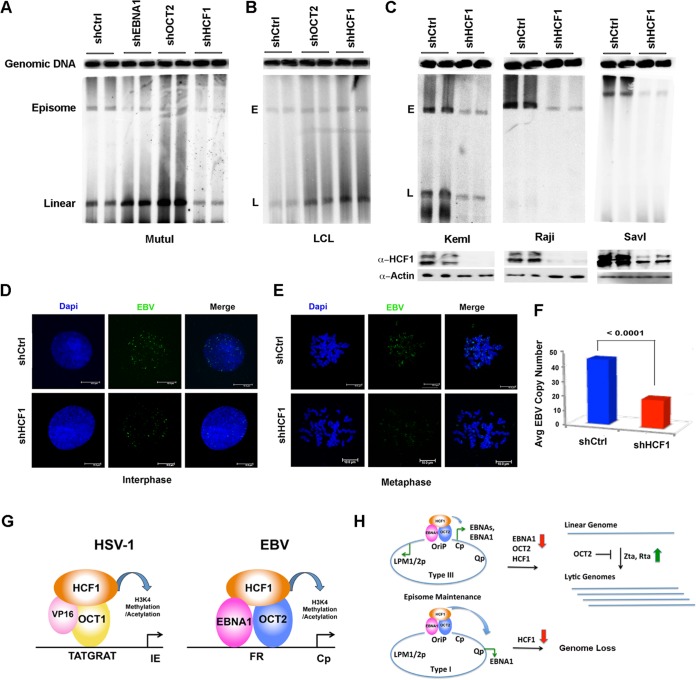
Depletion of HCF1 destabilizes EBV episomes. (A) PFGE analysis of MutuI cells transduced with shCtrl, shEBNA1, shOCT2, or shHCF1. The positions of cellular genomic DNA, viral episomal (E), and linear (L) DNA are indicated. (B) PFGE analysis of LCL cells transduced with shCtrl, shOCT2, or shHCF1. (C) PFGE analysis of KemI, Raji, or SavI cells transduced with shCtrl or shHCF1. Western blots for HCF1 and actin are shown in the bottom panels. (D) FISH analysis of EBV genomes in interphase MutuI cells transduced with shCtrl or shHCF1 and counterstained with 4′,6′-diamidino-2-phenylindole (Dapi). (E) Same as in panel D, except for mitotic cells. (F) Quantification of FISH analysis shown as representatives in panels D and E. (G) Model of HCF1 function as coactivator for EBNA1-OCT2 at FR resembling the HCF1 cofactor for VP16 and OCT1 at HSV IE genes. (H) Model of HCF1 function at OriP for transcription enhancer function and episome maintenance.

We next asked whether OCT2 or HCF1 had any effect on viral episome maintenance. We assayed EBV episome structure using PFGE and Southern blotting (Fig. 10). We first assayed shOCT2 and shHCF1 in MutuI cells (Fig. 10A). We found that shOCT2 induced a conversion of circular episomes to linear genomes, consistent with a trend to induce the lytic life cycle. A similar conversion was observed with shEBNA1 depletion, suggesting that these factors contribute to maintaining the episomal state of the latent genome. In contrast, HCF1 depletion led to a loss of both episome and linear forms of EBV. In LCLs, both shOCT2 and HCF1 led to an increase in linear forms of EBV, with little loss of episome (Fig. 10B). To further explore the role of HCF1 in regulating EBV episome maintenance, we assayed shRNA depletion in three EBV-positive BL cell lines, including Raji, which is incapable of lytic replication (Fig. 10C). Western blotting demonstrated an efficient depletion of HCF1 (Fig. 10C, bottom panels). We found that shHCF1 depletion led to a loss of EBV episomal genomes from these cells. To further validate these observations, we assayed the effect on shHCF1 on EBV genome maintenance by FISH. We found that shHCF1 led to an ∼3-fold loss of EBV genome FISH signal in both interphase and metaphase MutuI cells (Fig. 10D and E). Taken together, these results indicate that HCF1 has an important function in regulating EBV episome maintenance, especially in type I BL cells.

## DISCUSSION

EBV latency requires complex and dynamic regulation of viral gene transcripts. EBNA1 is essential for transcription activation of EBNA2 during primary infection of B lymphocytes, and OriP is known to function as an EBNA1-dependent transcriptional enhancer. In this study, we set out to understand how EBNA1 functions at the FR region to enhance transcription from viral promoters. We used several methods to confirm previous observations that OCT2 binds cooperatively with EBNA1 at the FR element *in vitro* and *in vivo*. We found that EBNA1 can also interact with HCF1 in an OriP-dependent manner. This interaction was dependent on the EBNA1 DNA binding domain and a region previously implicated in HAUSP7 interaction (42). Depletion of either OCT2 or HCF1 altered the histone modification patterns at OriP and at target promoters Cp and Qp, with a loss of H3K4me3 and H3 acetylation. This correlated with the loss of EBNA2 transcription in type III latency and EBNA1 transcription in type I latency. Furthermore, HCF1 depletion led to a striking loss of EBV genome maintenance in type I cells. Together, these findings indicate that OCT2 and HCF1 function in association with EBNA1 to maintain EBV latency by regulating OriP chromatin modifications and transcription enhancer function.

Histone H3K4me3 methylation is consistently elevated at the OriP region in the various cell types tested. The highest levels of H3K4me3 are typically found at the EBER transcripts (EBV coordinates 6629 to 7128) immediately upstream of FR (EBV coordinates 7421 to 8042). This peak is particularly pronounced in type I latency where EBER transcription is expressed at higher levels. The expansion of the H3K4me3 through OriP and into the Cp region is commonly observed in type III latency where Cp is active, while a more restricted peak of H3K4me3 is observed in MutuI cells where Cp is inactive. In an effort to identify factors that maintain this high level of H3K4me3 at OriP, we assayed various candidate proteins from the histone H3K4 methylase complexes, including HCF1. Of these candidates, we found that HCF1 was enriched at OriP and that it could interact with EBNA1 in the context of OriP. We also confirmed the previous observation that OCT2 can bind to the FR element cooperatively with EBNA1 (24, 25). We could show that purified OCT2 DBD bound cooperatively with EBNA1 DBD to FR oligonucleotides with two repeat elements. Since OCT2 did not bind efficiently to EBNA1 in coIP experiments and since HCF1 did not bind to EBNA1 without OriP in cell lysates, we suggest that HCF1 and OCT2 binding to FR is dependent on EBNA1. This configuration is reminiscent of the OCT1-VP16-HCF1 ternary complex found at HSV-1 immediate early gene promoters (Fig. 10G).

HCF1 has essential functions in host cell cycle chromatin and gene regulation. HCF1 is posttranslationally processed by proteolysis into amino- and carboxy-terminal peptides that have distinct functions (43). HCF1 does not possess any intrinsic DNA binding activity, but it interacts with chromatin-bound factors through its amino-terminal Kelch domain (44). We found that EBNA1 interacted primarily with the amino-terminal domain that included both the Kelch and basic domains. VP16 and other transcription factors bind predominantly to the HCF1 Kelch domain, while we found that the Kelch domain was not sufficient to interact with EBNA1. This suggests that EBNA1 interaction with HCF1 is different than several other known transcriptional coactivators. HCF1 has also been shown to have a function in maintaining chromosome structure, in part through binding to mitotic chromatin through its carboxy-terminal domain (45). We found that depletion of HCF1 resulted in the loss of EBV episomes from BL cells with type I latency, suggesting that HCF1 may also function in tethering EBV episomes to mitotic chromosomes in these cells. HCF1 was also required for production of EBNA1 mRNA. This is consistent with the well-characterized function of HCF1 in transcription regulation. In a previous study, we found that OriP forms a DNA loop with either Cp in type III latency or Qp in type I latency, corresponding to promoter activation status (21). Here, we show that histone modifications associated with enhancer function (H3K4me1 and H3K27ac) are enriched at OriP in both type I and type III latency (Fig. 2). The distribution of these histone modifications is different for each cell type, possibly reflecting the different ways that loops may be formed with target promoters. Histone modifications associated with promoter activation (H3K4me3 and H3K9ac) were enriched at Cp in type III latency and Qp in type I latency (Fig. 3). These epigenetic profiles were lost when HCF1 or OCT2 was depleted (Fig. 9). This suggests that OriP enhancer interactions are important for maintaining histone modifications at target promoters. We also observed that depletion of HCF1 led to an increase in heterochromatic H3K9me3 modification at Cp in type III latency. This is consistent with previous studies showing that HCF1 and associated histone demethylase LSD1 function to actively remove H3K9me3 prior to activation by MLL and H3K4me3 (46). For EBV latency, depletion of HCF1 and OCT2 can also lead to lytic reactivation, which affects histone modifications and transcription throughout the viral genome. We propose that HCF1 binding to OriP plays an important enhancer function by recruiting MLL histone methyltransferase complex. We speculate that the bipartite protein structure and mitotic chromatin association of HCF1 may help mediate the EBNA1-dependent DNA looping for transcription, and this may indirectly or directly regulate lytic reactivation and episome maintenance of EBV genomes.

EBNA1 interaction with HCF1 required the EBNA1 DBD along with amino acids 400 to 440. This region of EBNA1 is known to interact with HAUSP7 (42), and it is possible that the interaction of EBNA1 with HCF1 may be modulated by HAUSP7 binding or enzymatic function in deubiquitination. Further studies are required to understand whether additional EBNA1-interacting partners also associate with HCF1 or regulate its interaction with EBNA1. Given the central role of EBNA1 in regulating EBV latency, it is not surprising that it has numerous interaction partners that mediate its various functions in transcription, DNA replication, and episome maintenance.

Finally, a role for HCF1 and OCT2 in EBNA1-dependent transcription regulation raises important parallels with HSV-1 control of immediate early gene transcription. HSV-1 immediate early gene activation is critically dependent on VP16 and HCF1 functioning together, and cytoplasmic-nuclear shuttling of HCF1 regulates reactivation from latency in neurons (47, 48). HCF1 can be regulated through multiple mechanisms, and it is possible that some of these mechanisms regulate EBV latent and lytic cycle switches. Future studies are required to elucidate the molecular mechanism through which HCF1 and OCT2 are recruited to OriP and whether regulation of HCF1 controls EBV gene expression programs similar to that observed for HSV-1.
